# Electronic Cigarette Use among Mississippi Adults, 2015

**DOI:** 10.1155/2017/5931736

**Published:** 2017-08-16

**Authors:** Vincent L. Mendy, Rodolfo Vargas, Gerri Cannon-Smith, Marinelle Payton, Enkhmaa Byambaa, Lei Zhang

**Affiliations:** ^1^Office of Health Data and Research, Mississippi State Department of Health, Jackson, MS, USA; ^2^Department of Epidemiology and Biostatistics, School of Public Health, Jackson State University, Jackson, MS, USA; ^3^Center of Excellence in Minority Health and Health Disparities, Institute of Epidemiology and Health Services Research, School of Public Health, Jackson State University, Jackson, MS, USA; ^4^Department of Internal Medicine, University of California Davis, School of Medicine, Sacramento, CA, USA

## Abstract

Electronic cigarettes (e-cigarettes) are battery-powered devices that deliver nicotine in the form of aerosol. We identify differences and associations in e-cigarette use by sociodemographic characteristics and describe the reported reasons for initiating use among Mississippi adults. We used the 2015 Mississippi Behavioral Risk Factor Surveillance System, which collected information on e-cigarette use from 6,035 respondents. The prevalence of current e-cigarette use and having ever tried an e-cigarette was determined overall and by sociodemographic characteristics. Weighted prevalences and 95% confidence intervals were calculated, and prevalences for subgroups were compared using the *X*^2^ tests and associations were assessed using logistic regression. In 2015, 4.7% of Mississippi adults currently used e-cigarettes, while 20.5% had ever tried an e-cigarette. The prevalence of current e-cigarette use was significantly higher for young adults, whites, men, individuals unable to work, those with income $35,000–$49,999, and current smokers compared to their counterparts. Similar results were observed for having ever tried an e-cigarette. E-cigarette use was associated with age, race, income, and smoking status. Most (71.2%) of current e-cigarette users and over half (52.1%) of those who have ever tried e-cigarettes reported that a main reason for trying or using e-cigarettes was “to cut down or quit smoking.”

## 1. Introduction

Electronic cigarettes (e-cigarettes) are battery-powered devices that provide inhaled doses of nicotine by delivering a vaporized propylene glycol/nicotine mixture [1]. Carcinogens and toxins have been found in the aerosol of some e-cigarettes [2, 3]. E-cigarettes represent a major change in the tobacco control landscape [3]. They have been marketed as both a substitute for conventional cigarettes and a smoking cessation option by E-Cigarettes Companies [4, 5]; however, there is limited evidence that e-cigarette use promotes long-term smoking cessation [3, 6–9]. The Food and Drug Administration (FDA) recently finalized a rule extending its regulatory authority to all tobacco products, including e-cigarettes [2].

Recent evidence suggests that there has been an increase in the prevalence of e-cigarette use among adults in the United States (US) [10]. In Utah, for example, the use of e-cigarettes has increased fivefold in recent years, from 1.9% in 2011 to 10.5% in 2015 [11]. Given this trend, public health professionals are concerned that e-cigarette use could encourage smoking initiation, long-term dual use among current smokers, the reinitiation of smoking among former smokers, and the maintenance of nicotine addiction [2, 10]. In Mississippi, although the overall prevalence of current conventional cigarette use decreased relatively by 11.5% between 2011 (26.0%) and 2015 (23.0%), [12] the state still has one of the highest prevalences of current conventional smoking, and the prevalence is higher for some sociodemographic subgroups than for others [12]. Data on e-cigarette use among Mississippi adults are limited. In 2015, the Mississippi Behavioral Risk Factor Surveillance System (BRFSS) assessed e-cigarette use for the first time. We assess differences in e-cigarette use by sociodemographic characteristics, examine the association between e-cigarette use and sociodemographic characteristics and smoking status, and describe the reasons given for initiating use by select demographic characteristics among Mississippi adults.

## 2. Materials and Methods 

### 2.1. Data Source

We analyzed data from the 2015 Mississippi Behavioral Risk Factors Surveillance System (BRFSS), which included an optional module on e-cigarette use. The BRFSS is a state-based, random-digit-dialed telephone survey of the US noninstitutionalized civilian population aged 18 years or older. The survey is conducted in all 50 states, the District of Columbia, and three US territories (Puerto Rico, Guam, and the US Virgin Islands). Data from the BRFSS have been shown to reliably and validly assess health risk factors [13]. Beginning in 2011, BRFSS data included both landline and cell phone surveys and a new weighting methodology was used to improve accuracy of the data [14]. The BRFSS study has been approved by the human research review board at each state's department of health. Detailed information about BRFSS is available at https://www.cdc.gov/brfss/. This study was deemed exempt by the Mississippi State Department of Health Institutional Review Board.

### 2.2. E-Cigarette Use

E-cigarette use was determined by first describing an e-cigarette for the respondent (“The next questions are about electronic cigarettes, also known as e-cigarettes, vaping devices, or hookah pens. E-cigarettes look like regular cigarettes, but are battery-powered and produce vapor instead of smoke. E-cigarettes can be bought as one-time, disposable products, or can be bought as reusable kits with a cartridge. These cartridges come in many different flavors and nicotine concentrations. Using e-cigarettes is also called “vaping.”). Respondents were then asked “Have you ever tried an e-cigarette, even just one time in your entire life?” Those who answered “yes” were classified as having “ever tried an e-cigarette.” These respondents were then asked “Do you now smoke e-cigarettes every day, some days, or not at all?” Respondents who reported using e-cigarettes every day or some days were classified as current e-cigarette users. Respondents were then asked “What best describes your reason for using or trying e-cigarettes?” Possible responses were 1: to cut down or quit smoking, 2: I visit places that prohibit smoking, 3: for enjoyment or pleasure, 4: just tried it a few times, and 5: other [5, 15].

### 2.3. Sociodemographic Characteristics

Sociodemographic variables include age group (18–24, 25–44, 45–64, and ≥65 years), sex, race (black, white, and other races), education level (<high school, high school or equivalent, and >high school), employment status (employed, unemployed, student, retired, and unable to work), and annual household income (<$20,000, $20,000–$34,999, $35,000–$49,999, $50,000, and no answer).

### 2.4. Smoking Status

Respondents who reported smoking ≥ 100 cigarettes during their lifetime and smoking every day or some days at the time of the survey were classified as current cigarette smokers. Those who reported smoking ≥ 100 cigarettes during their lifetime but not smoking at the time of the survey were classified as former smokers. Respondents who reported smoking fewer than 100 cigarettes during their life time were classified as nonsmokers.

### 2.5. Statistical Analyses

Weighted prevalences and 95% confidence intervals (CI) were calculated. E-cigarette use was compared across sociodemographic characteristics using chi-square tests and the associations between e-cigarette use and sociodemographic characteristics were examined using logistic regression adjusted for age, sex, race, education, employment, income, and conventional smoking. SAS version 9.4 (SAS Institute, Inc., Cary, North Carolina) was used to perform all statistical analyses accounting for the complex sample design; significance levels were determined based on a *p* value less than 0.05.

## 3. Results and Discussion

### 3.1. Results

The mean age was 46.8 years; one-third (33.5%) were between the ages of 25 and 44 years; more than a third (35.3%) were black; over half (52.1%) were women and a similar proportion (51.4%) reported having more than a high school education; more than half (57.1%) reported being employed and about a quarter (24.7%) had an annual household income of less than $20,000 (Table 1).

In 2015, overall, 4.7% (95% CI 3.8–5.6) of Mississippi adults were current e-cigarette users, while one in five (20.5%, 95% CI 18.7–22.2) reported that they had tried e-cigarettes at least once. The reported prevalence of having ever tried an e-cigarette was significantly higher among those who are 18–24 years old (33.9%, 95% CI 26.5–41.3, *p* < 0.0001), whites (23.0%, 95% CI 20.8–25.3, *p* = 0.0014), men (24.4%, 95% CI 21.5–27.3, *p* < 0.0001), students [as employment status] (30.3%, 95% CI 20.3–40.2, *p* < 0.0001), those with an annual household income of less than $20,000 (24.5%, 95% CI 20.7–28.3, *p* = 0.0418), and current smokers (56.0%, 95% CI 51.5–60.4, *p* < 0.0001) than among their respective counterparts (Table 2). The prevalence of current e-cigarette use was significantly higher among young adults (18–24 years of age, 8.6%, 95% CI 4.5–12.7, *p* < 0.0001), whites (5.6%, 95% CI 4.4–6.8, *p* = 0.0190), men (5.7%, 95% CI 4.2–7.3, *p* = 0.0367), those unable to work (6.2%, 95% CI 3.7–8.8, *p* = 0.0019) those with an annual household income of $35,000–$49,000 (7.2%, 95% CI 3.9–10.5, *p* = 0.0376), and current smokers (14.7%, 95% CI 11.6–17.8, *p* < 0.0001) than among their respective counterparts (Table 3).

**Figure 1:**
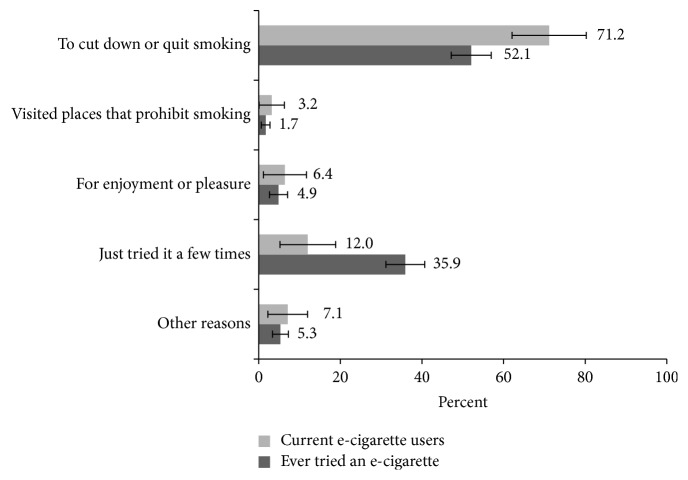
Reasons for trying or using e-cigarettes among Mississippi adults who have ever tried e-cigarettes and current e-cigarette users, Behavioral Risk Factor Surveillance System, 2015.

Among Mississippi adults who had tried e-cigarettes at least once, more than half (52.1%, 95% CI 47.2–57.0) and most (71.2%, 95% CI 62.1–80.3) of current e-cigarette users said that their main reason for using or trying e-cigarettes was “to cut down or quit smoking” (Figure 1). Only 7.1% of nonsmokers reported having ever tried an e-cigarette (Table 3); among this group, one in five (21.6%) said that the reason for trying or using e-cigarettes was “for enjoyment or pleasure” (Table 4). Among those aged 18–24 years, 17.1% reported trying or using e-cigarettes “for enjoyment or pleasure” (Table 4).

Based on regression models adjusted for age, sex, race, education, employment status, income, and smoking status, Mississippi adults aged 18–24 years (adjusted odds ratio [AOR] 10.1, 95% CI 3.4–29.6, *p* < 0.0001), 25–44 years (AOR 3.4, 95% CI 1.4–8.5, *p* = 0.0076), and 45–64 years (AOR 3.1, 95% CI 1.4–6.8, *p* < 0.0045) have significantly higher odds of current e-cigarette use compared to adults 65 years and older (Table 5). The odds of current e-cigarette use were significantly higher among white adults (AOR 2.0, 95% CI 1.2–3.4, *p* = 0.0127) compared to black adults and significantly higher among current (AOR 15.2, 95% CI 7.4–31.3, *p* < 0.0001) and former conventional cigarette smokers (AOR 3.5, 95% CI 1.5–8.0, *p* = 0.0032) compared to never smokers.

Similarly, the odds of ever tried an e-cigarette were significantly higher among Mississippi adults aged 18–24 years (AOR 15.9, 95% CI 8.7–28.9, *p* < 0.0001), 25–44 years (AOR 5.5, 95% CI 3.6–8.6, *p* < 0.0001), and 45–64 (AOR 3.3, 95% CI 2.3–4.8, *p* < 0.0001) years compared to adults 65 years and older. The odds of ever tried an e-cigarette were significantly higher among white adults (AOR 2.0, 95% CI 1.5–2.8, *p* < 0.0001) compared to black adults, significantly higher among those with an annual household income of less than $20,000 (AOR 1.6, 95% CI 1.0–2.4, *p* = 0.0348) compared to those with an annual household income of $50,000 or more, and significantly higher among current (AOR 21.8, 95% CI 14.9–31.6, *p* < 0.0001) and former conventional cigarette smokers (AOR 4.1, 95% CI 2.8–5.9, *p* < 0.0001) compared to never smokers (Table 5).

### 3.2. Discussion

To our knowledge, this is the first statewide study to assess e-cigarette use among adults in Mississippi. In 2015, 4.7% of adult Mississippians were current e-cigarette users and 20.5% had ever tried an e-cigarette. This prevalence is higher than the national prevalence: among US adults, 3.7% were current e-cigarette users and 12.6% had ever smoked an e-cigarette in 2014 [5]. The use of e-cigarettes differed significantly by age, race, gender, employment status, annual household income, and smoking status among Mississippi adults for both those who ever tried an e-cigarette and current e-cigarette users. When sociodemographic characteristics were controlled, current e-cigarette use was significantly associated with age, race, and smoking status among Mississippi adults. Similarly, ever tried an e-cigarette was significantly associated with age, race, income, and smoking status among Mississippi adults. This finding is consistent with previous studies [2, 5, 15, 16]. A recent online survey of over 17,000 US adults indicated that they have been widely exposed to e-cigarette marketing through the media, and that such marketing targeted specific demographic groups [4, 17, 18]. The targeted marketing of e-cigarettes to specific subgroups may explain sociodemographic differences in e-cigarette use among Mississippi adults, especially young adults. A greater awareness of e-cigarettes due to television advertising [18] and social media [19] as well as a perception that they are less harmful than traditional cigarettes may have led to a higher prevalence of e-cigarette use among young adults [20]. In addition, low cost and ease of accessibility are potential contributors to e-cigarettes use [16]. In 2015, the prevalence of current conventional smoking was 23.0% [12] among Mississippi adults; this prevalence is similar to the proportion of respondents who reported that they had ever tried an e-cigarette (20.5%). Based on these observations, public health professionals, advocacy organizations, and policymakers should be concerned about ongoing conventional smoking prevention and cessation efforts in the state. Future assessments should examine the impact of the targeted marketing of e-cigarettes among subgroups, especially young adults and current conventional smokers in Mississippi.

The current use of e-cigarettes was highest among current smokers (14.7%) and those aged 18–24 (8.6%). The same two groups were most likely to have ever tried e-cigarettes: more than half (56.0%) of current smokers and a third (33.9%) of respondents aged 18–24 reported having tried an e-cigarette at least once. In addition, 17.9% of former smokers, 7.1% of nonsmokers, and 30.3% of students reported having ever tried an e-cigarette. These findings highlight the need for e-cigarette policies in Mississippi, particularly policies focused on preventing young adults and nonsmokers from initiating e-cigarette use to preserve the previous gains in smoking prevention. Policy guidance on e-cigarettes from the American Heart Association promotes the inclusion of e-cigarettes in smoke-free laws, in state regulations that prohibit the sale of e-cigarettes to minors, and in laws that restrict the marketing and advertising of e-cigarettes to minors, as well as monitoring e-cigarettes use and taxation [3]. A recent national study reported an increase in e-cigarettes sales, from 2011 to 2015 [21]. Currently, Mississippi does not levy a tax on e-cigarettes [22]. While there are 124 smoke-free cities and towns in Mississippi, only 79 of these have ordinances that include restrictions on e-cigarettes [22]. Unregulated e-cigarette use has the potential to erode gains in conventional smoking cessation and smoke-free laws [3]. Evidence suggests that increasing retail prices and taxing e-cigarettes could lead to a reduction in sales [23].

Notably, two-thirds (66.8%) of current conventional cigarettes smokers who have ever tried e-cigarettes and most (82.5%) of current conventional cigarettes smokers who are current e-cigarette users reported that their main reason for using e-cigarettes was “to cut down or quit smoking”. However, the evidence on the use of e-cigarettes as a cessation aid is unclear [8]. Future studies should assess the efficacy of e-cigarettes as a smoking cessation aid or smoking substitute to prevent relapse among Mississippi smokers.

The most significant health issue related to e-cigarettes is whether or not e-cigarette use reduces the overall tobacco-associated health risk [3, 24]. Given the limited evidence on the health effects of e-cigarettes [9], there is a need for continual monitoring of e-cigarette use in Mississippi to assess awareness, prevalence, disparities, possible health effects and health promotion, and prevention strategies to reduce their use.

These findings have three main potential limitations. First, BRFSS consists of self-reported information on e-cigarette use, which is subject to recall bias and social desirability bias [25]; however, past studies have validated self-reported smoking data [26]. Second, because the e-cigarette module was only included in the Mississippi BRFSS in 2015, we could not assess trends in e-cigarette use over time [10]. Third, because the data are cross-sectional, we cannot make causal inferences based on the results. Finally, Mississippi BRFSS data include only adults (18 years and older); therefore, the findings may not be generalizable to younger populations. Key strengths of the current study include the use of a representative sample of the Mississippi adult population.

## 4. Conclusions

In 2015, about 5% of Mississippi adults were current e-cigarette users and one in five adults reported having ever tried an e-cigarette. E-cigarette use among adults differed by sociodemographic characteristics and by smoking status. E-cigarette use is associated with age, race, income, and smoking status among Mississippi adults. Most current e-cigarette users and more than half of those who have ever tried e-cigarettes reported that a main reason for trying or using e-cigarettes was “to cut down or quit smoking.” These findings highlight the need for e-cigarette policies and community interventions addressing the initiation of e-cigarette use among at-risk subgroups in Mississippi.

## Figures and Tables

**Table 1:** Sociodemographic characteristics of Mississippi adults, Behavioral Risk Factor Surveillance System, 2015.

Characteristic	%^a^ (*n* = 6,035)	95% CI
Age, y		
18–24	14.0	12.2–15.7
25–44	33.5	31.6–35.3
45–64	32.9	31.2–34.5
≥65	19.7	18.6–20.7
Race		
Black	37.1	35.3–38.9
White	59.6	57.7–61.4
Other races	3.3	2.5–4.2
Sex		
Male	47.9	46.1–49.8
Female	52.1	50.2–53.9
Education level		
<high school	18.5	16.9–20.2
High school or equivalent	30.0	28.3–31.7
>high school	51.4	49.6–53.3
Employment status		
Employed	57.1	55.1–59.1
Unemployed	15.0	13.5–16.6
Student	6.8	5.5–8.1
Retired	21.1	19.9–22.4
Unable to work	13.5	12.4–14.7
Annual household income ($)		
<20,000	24.7	23.1–26.4
20,000–34,999	22.0	20.4–23.6
35,000–49,999	10.7	9.5–11.9
≥50,000	26.7	25.1–28.3
No answer	15.9	14.5–17.3

CI, confidence interval; ^a^weighted percent.

**Table 2:** Ever tried an e-cigarette by sociodemographic characteristics among Mississippi adults, Behavioral Risk Factor Surveillance System, 2015.

Characteristic	Ever tried an e-cigarette^b^		
	%^a^ (*n* = 6,035)	95% CI	*p* value^c^
Overall	20.5	18.7–22.2	
Age, y			
18–24	33.9	26.5–41.3	<0.0001
25–44	26.3	22.8–29.7	
45–64	19.1	16.7–21.5	
≥65	5.1	3.9–6.3	
Race			
Black	15.7	12.9–18.5	0.0014
White	23.0	20.8–25.3	
Other races	22.8	10.6–34.9	
Sex			
Male	24.4	21.5–27.3	<0.0001
Female	17.0	14.9–19.0	
Education level			
<high school	21.3	16.6–26.1	0.1714
High school or equivalent	22.8	19.6–26.1	
>high school	18.9	16.7–21.1	
Employment status			
Employed	22.9	20.3–25.6	<0.0001
Unemployed	26.8	21.2–32.5	
Student	30.3	20.3–40.2	
Retired	7.2	5.5–9.0	
Unable to work	19.9	16.0–23.8	
Annual household income ($)			
<20,000	24.5	20.7–28.3	0.0418
20,000–34,999	21.3	17.5–25.1	
35,000–49,999	20.6	15.6–25.7	
≥50,000	17.1	14.0–20.1	
No answer	18.6	14.2–23.1	
Smoking status			
Current	56.0	51.5–60.4	<0.0001
Former	17.9	14.3–21.4	
Never	7.1	5.6–8.7	

CI, confidence interval; ^a^weighted percent; ^b^ever tried an e-cigarette were respondents who answered “yes” to “Have you ever tried an e-cigarette, even just one time in your entire life?”; ^c^determined by *X*^2^ test.

**Table 3:** Current e-cigarette use among Mississippi adults by sociodemographic characteristics, Behavioral Risk Factor Surveillance System, 2015.

Characteristic	Current e-cigarette users^b^		
	%^a^ (*n* = 6,035)	95% CI	*p* value^c^
Overall	4.7	3.8–5.6	
Age, y			
18–24	8.6	4.5–12.7	<0.0001
25–44	5.5	3.7–7.2	
45–64	4.9	3.6–6.1	
≥65	0.9	0.4–1.4	
Race			
Black	2.9	1.6–4.3	0.0190
White	5.6	4.4–6.8	
Other races	8.1	0.8–15.3	
Sex			
Male	5.7	4.2–7.3	0.0367
Female	3.8	2.8–4.8	
Education level			
<high school	4.4	2.4–6.5	0.1171
High school or equivalent	6.2	4.2–8.2	
>high school	4.0	2.9–5.1	
Employment status			
Employed	5.5	4.0–6.9	0.0019
Unemployed	6.0	3.1–8.9	
Student	3.7	0.1–7.3	
Retired	1.2	0.6–1.9	
Unable to work	6.2	3.7–8.8	
Annual household income ($)			
<20,000	6.2	4.1–8.3	0.0376
20,000–34,999	4.4	2.6–6.3	
35,000–49,999	7.2	3.9–10.5	
≥50,000	3.6	2.0–5.2	
No answer	2.9	1.3–4.5	
Smoking status			
Current	14.7	11.6–17.8	<0.0001
Former	3.3	1.6–4.9	
Never	1.1	0.4–1.8	

CI, confidence interval; ^a^weighted percent; ^b^current users were respondents who reported ever trying an e-cigarette and answered “every day or some days” to “Do you now smoke e-cigarettes every day, some days, or not at all?”; ^c^determined by *X*^2^ test.

**Table 4:** Reasons for trying or using e-cigarettes among Mississippi adults who have ever tried e-cigarettes by select demographic characteristics, Behavioral Risk Factor Surveillance System, 2015.

Characteristic	To try to quit smoking		For enjoyment or pleasure	
	%^a^ (*n* = 6,035)	95% CI	%^a^ (*n* = 6,035)	95% CI
Overall	52.1	47.2–57.0	4.9	2.6–7.1
Age (years)				
18–24	34.4	21.1–47.8	17.1	7.8–26.5
25–44	49.2	41.4–57.0	2.2	0.3–4.1
45–64	65.7	59.0–72.4	0.8	0.1–1.5
≥65	65.6	54.4–76.7	1.1	0.0–2.5
Race				
Black	49.4	39.5–59.3	6.8	1.8–11.9
White	52.4	46.7–58.2	3.9	1.5–6.3
Other races	65.8	36.0–95.5	10.4	0.0–26.9
Sex				
Male	45.9	38.9–52.8	4.9	1.7–8.2
Female	60.0	53.5–66.5	4.8	1.9–7.7
Smoking status				
Current	66.8	61.0–72.6	0.5	0.0–0.9
Former	49.9	38.6–61.2	1.9	0.0–4.9
Never	8.2	3.0–13.3	21.6	11.7–31.6

CI, confidence interval; ^a^weighted percent.

**Table 5:** Odds of current e-cigarette use and ever tried an e-cigarette among Mississippi adults by sociodemographic characteristics, Behavioral Risk Factor Surveillance System, 2015.

Characteristic	Current e-cigarette users^a^			Ever tried an e-cigarette^b^		
	AOR^c^	95% CI	*p* value	AOR	95% CI	*p* value
Age (years)						
18–24	10.1	3.4–29.6	<0.0001	15.9	8.7–28.9	<0.0001
25–44	3.4	1.4–8.5	0.0076	5.5	3.6–8.6	<0.0001
45–64	3.1	1.4–6.8	0.0045	3.3	2.3–4.8	<0.0001
≥65	1.0	Referent		1.0	Referent	
Race						
White	2.0	1.2–3.4	0.0127	2.0	1.5–2.8	<0.0001
Black	1.0	Referent		1.0	Referent	
Other races	2.5	0.6–9.5	0.1907	1.2	0.5–3.1	0.6497
Sex						
Female	1.0	Referent		1.0	Referent	
Male	1.2	0.8–1.9	0.3831	1.2	0.9–1.6	0.1126
Education level						
<high school	0.7	0.4–1.4	0.3413	0.8	0.5–1.2	0.2609
High school or equivalent	1.2	0.7–1.9	0.5919	1.0	0.8–1.4	0.8139
>high school	1.0	Referent		1.0	Referent	
Employment status						
Employed	1.0	Referent		1.0	Referent	
Unemployed	0.7	0.4–1.4	0.3761	0.9	0.6–1.4	0.6852
Student	0.6	0.2–1.8	0.3462	1.3	0.7–2.6	0.4707
Retired	0.7	0.3–1.5	0.3356	0.8	0.5–1.2	0.2767
Unable to work	1.3	0.7–2.3	0.4479	0.8	0.5–1.2	0.2196
Annual household income ($)						
<20,000	1.5	0.7–3.1	0.3033	1.6	1.0–2.4	0.0348
20,000–34,999	1.0	0.5–2.1	0.9369	1.2	0.8–1.8	0.383
35,000–49,999	1.8	0.8–3.8	0.1298	1.1	0.7–1.7	0.7367
≥50,000	1.0	Referent		1.0	Referent	
No answer	0.8	0.4–1.9	0.639	1.3	0.8–2.1	0.3258
Smoking status						
Current	15.2	7.4–31.3	<0.0001	21.8	14.9–31.6	<0.0001
Former	3.5	1.5–8.0	0.0032	4.1	2.8–5.9	<0.0001
Never	1.0	Referent		1.0	Referent	

AOR, adjusted odds ratio; CI, confidence interval; ^a^current users were respondents who reported ever trying an e-cigarette and answered “every day or some days” to “Do you now smoke e-cigarettes every day, some days, or not at all?”; ^b^ever tried an e-cigarette were respondents who answered “yes” to “Have you ever tried an e-cigarette, even just one time in your entire life?”; ^c^adjusted for age, sex, race, education, employment status, income, and smoking status.
